# Amyloidosis in a child with Hyperimmunoglobulin D syndrome

**DOI:** 10.1186/1546-0096-9-S1-P2

**Published:** 2011-09-14

**Authors:** Ruhan Dusunsel, Zubeyde Gunduz, Funda Bastug, Ismail Dursun, Hakan M Poyrazoglu, Sibel Yel, Sebahat Tulpar, Kemal Deniz, Erkan Demirkaya

**Affiliations:** 1 Erciyes University Faculty of Medicine Department of Pediatric Nephrology and Rheumatology, Turkey; 2 Department of Pathology, Kayseri, Turkey; 3 Gulhane Military School Department of Pediatric Nephrology and Rheumatology, Ankara, Turkey

## Background and aim

Amyloidosis in children autoinflammatory disease is one of the most important unfavorable outcomes.

## Case

A-7 year old boy was admitted with complaints of edema on his eyelid and lower extremity. On admission, he had edema, hepatosplenomegaly, proteinuria and hypoalbuminemia. So his diagnosis was accepted as nephrotic syndrome and steroid therapy was started. Since he did not respond steroid therapy, kidney biopsy was performed. Biopsy findings were consistent with amyloidosis. Steroid therapy was ceased and colchicine was started. The patient was reevaluated for autoinflammatory diseases. His parent told that he had slightly periodic fever and he had no abdominal pain, arthritis, pleuritis and erysipelas-like erythema. MEFV mutation was normal. Other autoinflammatory syndromes were investigated and he had elevated serum IgD concentration. Mevalonate kinase gene mutation was positive for G326R/V377I. His diagnosis was Hyperimmunoglobulin D syndrome (HIDS). Having a poor response to colchium therapy, anti-TNF therapy (etanercept) was planned.

## Conclusion

Amyloidosis in children with HIDS was rarely reported. In literature, we present the first report of the occurrence of renal AA amyloidosis causing severe nephrotic syndrome, in a Turkish children affected with HIDS.

